# Animal-vehicle collisions, roadkilled animals, and human health: A scoping review protocol

**DOI:** 10.1371/journal.pone.0341107

**Published:** 2026-08-04

**Authors:** Fiona C. Doherty, Travis R. Scheadler, Nadia Matin

**Affiliations:** 1 College of Social Work, University of Tennessee Knoxville, Nashville, Tennessee, United States of America; 2 College of Health and Human Services, School of Social Work, University of North Carolina at Charlotte, Charlotte, North Carolina, United States of America; MARE – Marine and Environmental Sciences Centre, PORTUGAL

## Abstract

**Background:**

Animal-vehicle collisions (AVCs) are a growing global problem with significant implications for human, animal, and ecosystem health. In the United States, approximately one million vertebrates are killed by vehicles each day. Research on roadkilled animals has increased over time, especially regarding wildlife and ecosystem impacts, economic costs, and human physical health risks. However, less is known about how involvement with AVCs or encounters with roadkilled animals influences human health more broadly, including psychological, emotional, psychosocial, and community-level outcomes.

**Objectives:**

Guided by the One Health framework, this study protocol presents the methodological plan to identify and synthesize current evidence on how AVCs and exposure to roadkilled animals affect human health. The scoping review has not yet been conducted.

**Search strategy:**

The methodological approach follows PRISMA-ScR guidelines and Arksey and O’Malley’s five stage framework. A comprehensive, librarian‑assisted search will be conducted across six databases—Scopus, CABI Digital Library, Web of Science, Wildlife & Ecology Studies Worldwide, TRID, and PubMed—without restrictions on publication year or peer‑review status. Reference lists of included records will also be screened. Three reviewers will independently screen the titles, abstracts, and full texts, with each record reviewed by a combination of two reviewers.

**Eligibility criteria:**

Records will be included if they examine how AVCs or roadkilled animals affect any dimension of human physical, mental, or social well‑being and are available in English.

**Charting methods:**

Data will be extracted using a standardized form and analyzed through content analysis to describe the distribution of evidence and organize findings into themes.

**Conclusions:**

By consolidating dispersed knowledge across disciplines, this review will provide the first comprehensive overview of how AVCs and roadkilled animal exposure affect human health. Findings will inform public policy, transportation planning, wildlife management, and future interdisciplinary research aimed at integrated One Health solutions.

## Introduction

In the United States (U.S.), approximately one million vertebrates are killed by animal-vehicle collisions (AVCs) every day, while in Europe, 194 million birds and 29 million mammals are killed annually [1,2]. Despite imperfect tracking systems, existing data show an upward trend associated with urbanization and traffic density, with mammal deaths from AVCs rising fourfold over the past fifty years [3,4]. The increasing prevalence of AVCs poses interconnected risks to animal, ecosystem, and human health [1,5].

Research attention to roadkilled animals—those struck and killed by vehicles—has increased in recent years, particularly in studies examining wildlife and ecosystem impacts, human injury or fatality risks, economic costs, and mitigation strategies [1,5–7]. AVCs reduce wildlife population sizes, diminish genetic diversity, and compromise ecosystem structure and balance [1]. Road mortality is one of the leading sources of anthropogenic mortality for many vertebrates and the single greatest cause of death for approximately 28% of studied populations [8]. Beyond direct mortality, roads fragment the habitats and movement of animals, contributing to population isolation and subsequently higher local extinction risk [6,8]. Roadkilled animals can also serve as hosts for environmental contaminants and disease, posing additional possible physical health risks and stressors distinct from the collision event itself [9]. These impacts are not limited to ecological systems. In the U.S., AVCs result in over 440 human fatalities and 59,000 injuries annually, along with an estimated economic cost of over $8 billion per year [1,10].

Greater attention is needed to understand how involvement with AVCs and exposure to roadkilled animals affect other dimensions of human health, including psychological, emotional, psychosocial, and community-level outcomes [1,3,11]. Research indicates that up to 25% of patients severely injured in AVCs experience psychological reactions, including 3-6% that develop long-term post-traumatic stress disorder [1]. Impacts also extend to wildlife professionals, who report grief when responding to AVC incidents [11]. Even indirect exposure can affect well-being, as tourists in Tasmania reported sadness, anger, and disgust when encountering roadkilled animals, which impacted their likelihood of repeat tourism [12]. Women participants reported greater emotional distress, highlighting the need for demographic comparisons within the exploration of AVCs and human health. As evidence develops, increased scholarly attention is warranted to examine the role of roadkilled animals within human-animal relations and well-being.

This study—a scoping review that has not yet been conducted—aims to identify and synthesize existing research on how AVCs and encounters with roadkilled animals affect human health. Prior reviews have examined AVCs and roadkilled animals broadly, including syntheses of road ecology (e.g., wildlife impacts, mitigation strategies) and bibliometric analyses of study locations, species, topics, and methodologies [1,5]. However, no review to date has specifically focused on human health outcomes. To address this gap, we employ a tailored scoping review methodology, which is well-suited to the breadth of the research question [13], and a search strategy designed to identify evidence linking AVCs and roadkilled animal exposure to human health.

We operationalize health using the World Health Organization (WHO) definition, which includes the physical, mental, and social dimensions of well-being [14,15]. Grounded in a One Health framework, which emphasizes the interdependence of human, animal, and ecosystem health [16], this review examines the interconnected health dimensions that converge at the road interface. By consolidating this evidence base, the review aims to inform public policy, guide infrastructure planning, and support the development of integrated, transdisciplinary interventions for human, ecosystem, and animal well-being.

## Methods

This paper presents the protocol for a scoping review that has not been pre-registered. Notably, a scoping review is an appropriate methodology for this proposed study given the broad nature of the research question and inclusion criteria [13], described below. The Preferred Reporting Items for Systematic Reviews and Meta-Analyses Extension for Scoping Reviews (PRISMA-ScR) Checklist was followed to design this study (see S1 File). Arksey and O’Malley’s framework for scoping reviews was also used as a guide [17]. Their framework involves five stages: (a) identifying the research question, (b) identifying relevant studies, (c) study selection, (d) charting the data, and (e) collating, summarizing, and reporting the results. A university librarian was consulted to help ensure that this design is appropriate and feasible.

### Identifying the research question

The first stage in Arksey and O’Malley’s framework involves the identification of the research question [17]. To be consistent with their recommendations, scoping review questions should be broad and should incorporate several key aspects. More specifically, the research question was developed based on the PCC (population, concept, context) framework for scoping reviews. For this study, the population will be humans. Much of the literature on AVCs and roadkilled animals has emphasized wildlife conservation efforts, physical human injury, and economic impact [1,5,10]. This scoping review will focus on collating literature that has examined the direct influence of AVCs and roadkilled animals on humans. Second, the concept in this study will be the physical, mental, and social well-being impacts, at the individual or community level, following interaction between humans and roadkilled animals, which may include hitting an animal with a motor vehicle, seeing or disposing of a roadkilled animal, or other possible behaviors. Finally, the context for this study includes roads, sidewalks, and other transportation infrastructure in rural, urban, and suburban communities. Therefore, this review will attempt to answer the following question: How does the interaction with AVCs and roadkilled animals influence health among humans?

### Identifying relevant studies

Six databases will be used to identify potentially relevant records for inclusion. Specifically, Scopus, CABI Digital Library, Web of Science, Wildlife & Ecology Studies Worldwide, TRID, and PubMed will each be used to search for records related to the interactions between roadkilled animals and humans. The reference lists of included records will also be screened to identify records, as needed. Additionally, to remain broad and ensure the comprehensiveness of this scoping review, there will be no restrictions related to publication date or peer-review status. In other words, records—including peer-reviewed articles, chapters, grey literature, and other sources—from any time point will all be considered for inclusion. Indeed, a preliminary search determined that including records from various time periods and non-peer-reviewed records could strengthen the impact of this review.

Key terms that will be input into the six databases are based on how prior scholars and journals have described the phenomena of interest. Table 1 organizes the search terms by the core concepts of 1) AVCs and roadkilled animals and 2) human health and well-being. Boolean terms (e.g., and, or) and other appropriate syntax (e.g., asterisks, quotation marks) will be applied to the searches. The search strategy will include key terms, Boolean operators, and appropriate syntax across all fields. The search strategy is available in S2 File. The scoping review search was completed on January 6; subsequent steps, such as record screening and data extraction, have not yet begun.

**Table 1:** Search terms organized by core concept.

Animal-vehicle collisions and roadkilled animals	Human health and well-being
roadkill	health
road-killed animals	well-being
roadkilled animals	wellbeing
wildlife-vehicle collision	psychological
wildlife vehicle collision	psychosocial
animal-vehicle collision	mental health
animal vehicle collision	emotion
vehicular wildlife mortality	social-emotional
wildlife road mortality	social health
	social well-being
	social wellbeing
	community health
	community well-being
	community wellbeing
	distress
	stress
	grief
	grievance
	grieve
	sad
	despair
	trauma
	fear
	shame
	disgust
	hurt
	harm
	physical activity

### Study selection

Identified records will be uploaded to Covidence (https://www.covidence.org/), an online management software for reviews. Covidence, which has a free trial version, will be used as a tool to organize the records and automatically remove duplicates, but will not be used to conduct analyses or make decisions for the research team. Indeed, each of these steps can be replicated by other research teams without the use of Covidence. For example, researchers performing scoping reviews without Covidence may manually check all records to identify and remove duplicates.

Afterwards, the titles and abstracts of each included record will each be independently screened by two of three reviewers. Full-texts of records that passed the title and abstract screening but are unavailable in the universities’ library systems at the authors’ institutions will be retrieved by contacting the author(s) of the record via email. However, records will be excluded if the author(s) of the record are unresponsive to requests or if the record is otherwise inaccessible. Then, the full-texts of articles that passed the title and abstract screening will be similarly independently screened by two of three reviewers. At each stage, the three reviewers will meet to discuss and resolve any discrepancies. Specifically, the three authors will discuss all discrepancies until a unanimous decision has been reached. The study selection process will be guided by the inclusion and exclusion criteria listed below. The research team anticipates record screening to be completed in March 2026.

#### Inclusion and exclusion criteria.

Records will be included in the study if they meet the following criteria: 1) examine the influence of AVCs or roadkilled animals on the physical, mental, or social well-being of one or more human participants; 2) are available in English; 3) are published in any year; 4) report research conducted in any geographic region; 5) are published as peer-reviewed journal articles or grey literature; and 6) use quantitative, qualitative, or mixed methods designs. Records will be excluded if they: 1) do not include human participants; 2) do not examine the impact of AVCs or roadkilled animals on human well-being; or 3) are theoretical or conceptual articles, including reviews. A PRISMA flow diagram will be generated to depict the results from the record identification and screening processes.

### Charting the data

Data will be charted via a standardized extraction form, displayed in S3 File. The data from each included record will be extracted by one reviewer. Then, another reviewer will read and validate the extractions. The three reviewers will meet to discuss and resolve any discrepancies. The research team anticipates data extraction to be completed in April 2026.

### Collating, summarizing, and reporting the results

As recommended by Arksey and O’Malley, extracted data will be analyzed in two ways [17]. First, data will be analyzed via a narrative approach that describes the distribution of records based on geographic location and key outcomes. Second, data will be organized into themes, and key findings will be described.

Basic content analysis will be performed to facilitate this process [18–20]. Due to the broad nature of the research aim and the dearth of research in this area, an inductive coding approach will be used. Indeed, as recommended by Pollock et al., an inductive approach to coding in scoping reviews is best when the goal of the study is to develop a broad understanding of existing research in a given area [20]. As such, a codebook will not be created in order to preserve an inductive coding approach. Using a predefined codebook could constrain analysis by requiring data to fit pre-existing codes, which could prevent the research team from capturing nuanced or unexpected data [20].

This approach will involve multiple phases of coding. First, reviewers will re-familiarize themselves with the data by reading and re-reading the extracted content. Then, data from the key findings section of the extraction form will be coded inductively and independently by two reviewers. Two reviewers will use open coding to code the extracted data. Afterward, the three reviewers will meet to discuss the codes and resolve any discrepancies. Specifically, discrepancies will be resolved through conversation until a consensus is reached. Next, the three reviewers will independently sort the codes into categories and subcategories based on their similarities and differences. Throughout this process, numerical counts of codes will be used to help determine key findings and research gaps related to the research question in this review. The reviewers will then meet again to discuss and finalize the categories and subcategories. The reviewers will return to the included records to confirm that the categories and subcategories accurately reflect the data. Revisions to the categories and subcategories will be made, if necessary. Finally, the categories and subcategories will be described textually and depicted via tables and/or figures.

Note, though, that the reporting of the results will only involve a description of the key findings and will not involve a synthesis of key findings. This decision aligns with best practices for performing a scoping review [13,20]. Relatedly, in alignment with the purposes of a scoping review, a critical appraisal of the evidence will not be conducted [13,20]. The research team anticipates results to be completed in June 2026.

### Discrepancies between the protocol and final review

Any discrepancies between this protocol and the final version of the scoping review will be noted. If discrepancies occur, a rationale will be provided.

## Discussion

This scoping review will be the first to summarize the current knowledge base on AVCs, roadkilled animals, and human health, providing a centralized understanding of this overlooked dimension of human-animal relations. Guided by the One Health framework, the review will contextualize findings across environmental, animal, and human domains to inform transdisciplinary policy recommendations and potential interventions related to transportation planning, public health, recreation science, and ecology. The review will also inform future research by highlighting gaps in methodology, geographic representation, and sample diversity. The review team includes social work, philosophy, environmental justice, and exercise psychology professionals invested in developing integrated solutions for equitable and sustained human, environmental, and animal health. Potential pathways for future research include conducting empirical investigations of the mental and social health impacts of roadkilled animals, including demographic and cross-cultural comparisons.

## Supporting information

S1 File Preferred Reporting Items for Systematic Reviews and Meta-Analyses Extension for Scoping Reviews (PRISMA-ScR) Checklist. (PDF)

S2 File Search strategy. (DOCX)

S3 File Standardized extraction form. (DOCX)
