# Supplementary material for: Animal-vehicle collisions, roadkilled animals, and human health: A scoping review protocol
Source: PLoS One. 2026 Aug 4;21(8):e0341107. doi: 10.1371/journal.pone.0341107 (PMC13436736; doi:10.1371/journal.pone.0341107)
Supplement: Supplementary file 2 [file pone.0341107.s002.docx]

**S2 File: Search Strategy.**

**Search String (Search within all fields; no date range):**

((roadkill* OR “road-killed animal*” OR “roadkilled animal*” OR “wildlife-vehicle collision*” OR “wildlife vehicle collision*” OR “animal-vehicle collision*” OR “animal vehicle collision*” OR “vehicular wildlife mortality” OR “wildlife road mortality”)

AND

(health OR psychosocial OR psychological OR “mental health” OR emotion* OR “social-emotional” OR “well-being” OR wellbeing OR distress OR stress OR grief OR griev* OR sad* OR despair OR trauma* OR fear OR shame OR disgust OR hurt* OR harm* OR “physical activity” OR “community health” OR “community well-being” OR “community wellbeing” OR “social health” OR “social well-being” OR “social wellbeing”))

**Databases:**

- Scopus
- CABI Digital Library
- Web of Science
- Wildlife & Ecology Studies Worldwide
- TRID
- PubMed
