# Supplementary material for: Animal-vehicle collisions, roadkilled animals, and human health: A scoping review protocol
Source: PLoS One. 2026 Aug 4;21(8):e0341107. doi: 10.1371/journal.pone.0341107 (PMC13436736; doi:10.1371/journal.pone.0341107)
Supplement: Supplementary file 3 [file pone.0341107.s003.docx]

**S3 File: Standardized Extraction Form.**

The following information will be extracted from the included records:

1. Study ID number
2. Author name(s)
3. Article title
4. Publication year
5. Name of team member completing extraction
6. Name of team member reviewing extraction
7. Publication type: Peer-reviewed or grey literature
8. Study discipline
9. Geographic origin of data
10. Funding (if applicable)
11. Aim(s)/purpose
12. Theoretical framing (if applicable)
13. Sample size and demographics
14. Methodological design type: Quantitative, qualitative, mixed methods
15. Recruitment strategy
16. Data collection approach
17. Data analysis approach
18. Relevant findings
19. Sub-relevant findings
20. Proposed limitations
21. Unidentified limitations
22. Proposed future directions
23. Other notes
