# Supplementary material for: Animal-vehicle collisions, roadkilled animals, and human health: A scoping review protocol
Source: PLoS One. 2026 Aug 4;21(8):e0341107. doi: 10.1371/journal.pone.0341107 (PMC13436736; doi:10.1371/journal.pone.0341107)
Supplement: Supplementary file 4 [file pone.0341107.s005.docx]

**Response to Reviewers:**

Thank you for the opportunity to revise our manuscript. We greatly appreciate the reviewers’ thoughtful and constructive feedback, which has helped us strengthen the clarity, precision, and overall contribution of the paper. We have carefully considered and addressed all comments and suggestions, making revisions throughout the manuscript where appropriate. Detailed responses to each reviewer’s feedback are outlined below, with corresponding changes noted in the text.

**Editor:**

| **Editor Comment** | **Authors’ Response** |
| --- | --- |
| 1. Please ensure that your manuscript meets PLOS ONE's style requirements, including those for file naming. The PLOS ONE style templates can be found at  <https://journals.plos.org/plosone/s/file?id=wjVg/PLOSOne_formatting_sample_main_body.pdf> and  [https://journals.plos.org/plosone/s/file?id=ba62/PLOSOne_formatting_sample_title_authors_affiliations.pdf](https://track.editorialmanager.com/CL0/https:%2F%2Fjournals.plos.org%2Fplosone%2Fs%2Ffile%3Fid=ba62%2FPLOSOne_formatting_sample_title_authors_affiliations.pdf/1/010f019eb6413f75-6f264df3-34ca-4959-b6a8-db942026c23b-000000/Vm9cmT9EkS-Em4sW6XXuQ35vuGaiom2_jOwYhFThGZk=258) | We have reviewed the journal’s style requirements and updated our submission accordingly. |
| 2. When completing the data availability statement of the submission form, you indicated that you will make your data available on acceptance. We strongly recommend all authors decide on a data sharing plan before acceptance, as the process can be lengthy and hold up publication timelines. Please note that, though access restrictions are acceptable now, your entire data will need to be made freely accessible if your manuscript is accepted for publication. This policy applies to all data except where public deposition would breach compliance with the protocol approved by your research ethics board. If you are unable to adhere to our open data policy, please kindly revise your statement to explain your reasoning and we will seek the editor's input on an exemption. Please be assured that, once you have provided your new statement, the assessment of your exemption will not hold up the peer review process. | Thank you for this guidance, and we apologize for any confusion. This manuscript presents a protocol. Therefore, no study data have been generated and there are no data available for sharing at this protocol stage. However, we plan to make the data extraction form available as a supplemental file upon submission of the completed scoping review. |
| 3. Please include captions for your Supporting Information files at the end of your manuscript, and update any in-text citations to match accordingly. Please see our Supporting Information guidelines for more information: [http://journals.plos.org/plosone/s/supporting-information](http://track.editorialmanager.com/CL0/http:%2F%2Fjournals.plos.org%2Fplosone%2Fs%2Fsupporting-information/1/010f019eb6413f75-6f264df3-34ca-4959-b6a8-db942026c23b-000000/gY545Uzy6n3Ll2paJV0aFDPAyAycdqXLIsg4bSuXfqk=258).  If the reviewer comments include a recommendation to cite specific previously published works, please review and evaluate these publications to determine whether they are relevant and should be cited. There is no requirement to cite these works unless the editor has indicated otherwise. | Supporting information captions and in-text citations have been updated to match the journal guidelines. |
| 4. Please review your reference list to ensure that it is complete and correct. If you have cited papers that have been retracted, please include the rationale for doing so in the manuscript text, or remove these references and replace them with relevant current references. Any changes to the reference list should be mentioned in the rebuttal letter that accompanies your revised manuscript. If you need to cite a retracted article, indicate the article’s retracted status in the References list and also include a citation and full reference for the retraction notice. | The reference list has been checked and updated for accuracy. Four additional citations have been added per reviewer suggestion. |

**Reviewer 1:**

| **Reviewer Comment** | **Authors’ Response** |
| --- | --- |
| 1.1 The manuscript describes a scoping review protocol for understanding the various kinds of impacts of wildlife-vehicle collisions on humans. This is a relatively understudied aspect of roadkill, and I congratulate the authors for devising this study. I look forward to reading the results of the completed study, as it potentially has important repercussions for human health, and can also help argue for safer roads for wildlife.  The review protocol is well-written, and well-described. I have a few comments, specifically pertaining to the flow and readability of the text. Certain things need to be described and clarified, for the manuscript to be understood by the wider scientific readership. | Thank you for the kind and encouraging feedback. |
| 1.2 Abstract, Background: include physical (injury, fatality) | We now include physical health in the background section of the abstract. |
| 1.3 Abstract, Search strategy: I suggest detailing the process of managing screening (in the methods in detail) instead of prescribing a paid software, which may not be accessible to some researchers trying to replicate the process. | The mention of Covidence has been removed from the Abstract. We now provide greater detail about the screening process. More detail about the screening process has also been added to the Methods section (Pages 8-9). |
| 1.4 Page 4, para 1,2: Are you including domestic animal collisions too? It would be prudent to specify this at the beginning (since you also use the term ‘roadkilled animals’). Although the term ‘WVC’ indicates the study is concentrated on wildlife species only, a lot of domestic/free-ranging animals are also killed on roads, and can affect humans similarly. Alternately, define WVC at the beginning (wild animal fatalities resulting from collisions with vehicles). | We have revised the manuscript to use the broader term animal–vehicle collisions (AVCs) to encompass collisions involving all animals, including both wildlife and domestic species. This clarification has been incorporated in the manuscript’s title and throughout the manuscript for consistency. |
| 1.5 Page 4, Para 2: I suggest reorganising the second paragraph to direct the narrative towards human-impacts of WVCs, from the relatively well-studied aspect i.e., ecological impacts. | We agree that centering the discussion on human impacts is important. We considered reorganizing the paragraph accordingly; however, in response to Reviewer 2’s request for additional context on ecological impacts, we retained this material to acknowledge the interconnections between ecological and human health outcomes. To balance these perspectives, we have limited this discussion to a single paragraph and ensured that the broader literature review remains primarily focused on human health impacts. (Pages 3-4) |
| 1.6 Page 5, para 2: “prior reviews have examined WVCs and roadkilled animals more broadly” suggest defining ‘broad’ (mainly ecological, patterns etc). | We have revised the text to more clearly specify what is meant by “broad.” In particular, we note that prior reviews have taken a wide-ranging approach, including a comprehensive synthesis of road ecology (e.g., wildlife impacts, behavioral adaptations, spatiotemporal patterns, mitigation strategies, and research gaps) as well as bibliometric analyses mapping study locations, species, topics, and methodologies. This clarification has been added to improve readability. (Pages 4-5) |
| 1.7 Page 5, Paras 2,3: I understand the rationale behind the review (to be conducted) and how the scoping process is designed to achieve that. I believe separating the two would make for easier reading and comprehension for readers, and strengthen the introduction. I suggest: first state the merits/novelty of the proposed review framework, and then the perceived benefits of the resulting review results (or vice versa). | We have reorganized this section to first emphasize the novelty and methodological approach of the proposed review, followed by a clearer articulation of the expected contributions and benefits of the findings. (Pages 4-5) |
| 1.8 Page 8 (Study Selection): same comment regarding the software. Consider excluding the name, except if it is central to your review process, or no other alternatives exist (including open-source or free ones). | Covidence is a commonly used software for organizing records in systematic and scoping reviews and is widely recommended for its efficiency. Nevertheless, we added language to emphasize how the methods outlined in this protocol can be replicated without Covidence (Page 8) |
| 1.9 Page 8 (inclusion and exclusion criteria): “Records may be cross-sectional or longitudinal.” needs clarification | This sentence has been removed to reduce confusion as it is standard practice for scoping reviews to include cross-sectional and longitudinal studies. (Page 9) |
| 1.10 Page 10: Please describe the rationale behind independent coding of findings by reviewers, and not based on agreed upon codes and pre-defined categories. If I understand correctly, ‘open coding’ means it is up to the reviewers to interpret and code findings independently. On what basis will the discrepancies be resolved? Are the categories and sub-categories pre-defined? This section needs a little clarification and restructuring. Can the authors define what ‘code’ and ‘categories’ mean (maybe through examples) for readers unfamiliar with content analysis? | Thank you for your thoughtful questions. In response, we have added clarifying language throughout this section to better describe the coding process and articulate our rationale, drawing on established guidance from scoping review experts. (Page 10) |

**Reviewer 2:**

| **Reviewer Comment** | **Authors’ Response** |
| --- | --- |
| 2.1 This manuscript describes a scoping protocol (to assess the literature landscape, rather than to systematically review it) to assess the impacts of wildlife-vehicle collisions and exposure to roadkill on various factors of human health. The authors will be focusing on physical, social, and emotional impacts in their review. The protocol follows PRISMA-SrC for reviews and the results will be descriptive of the distribution of the literature rather than a systematic review of what the literature describes (as is appropriate for a scoping review). I believe that the proposed study would be important and of interest to sociologists, ecologists, and policymakers alike. The protocol is generally thorough and includes most of the necessary components (some are discussed by the authors but were not included in the submission). The citations all appear to be sound and properly formatted. | Thank you for the thoughtful review and encouraging feedback. |
| 2.2 I am a little surprised at the sparseness of the citations regarding the ecological consequences of roadkill given the ubiquity and significance of roadkill around the world. I am not a roadkill expert by any means, but even a cursory search yields several other ecological reviews that seem relevant to the problem at hand. For example (amongst many others):  https://doi.org/10.1016/j.jtrangeo.2006.11.006  https://doi.org/10.1007/s10344-019-1357-4  https://doi.org/10.1111/brv.12942  https://doi.org/10.1371/journal.pone.0166941  I understand that the purpose of the proposed review is to focus on the human impacts (especially the psychological/emotional impacts) rather than ecological factors. However, I feel that a stronger engagement with the ecological literature would benefit the authors regarding the various avenues through which roadkill yields emotional impacts (and thus the various ways of categorizing their findings in a review). For example, roadkill may also be a vector for contaminants/disease which (1) could be a significant physical impact of interacting with roadkill and (2) creates an entirely different collection of stressors than those caused by an initial collision.  Especially given that the authors intend to use an open coding approach, the more familiarity and understanding of the possible categories various studies may fall under, the better. I would be far more confident in the eventual choices of the authors if there was a clearer demonstration of engagement with the literature at this stage. | We agree that additional citations on the ecological consequences of roadkill would strengthen the illustration of its prevalence. Accordingly, we have incorporated four additional references to enhance the ecological literature and highlight a broader range of impacts, including the role of roadkilled animals as sources of contaminants and disease. (Page 3) |
| 2.3 If the scoping search has already been conducted (which is the case here), then I believe all of the search strings, their relevant databases, and the results at the time of the search should be reported, rather than the one example given in Supplemental File 2. If the string in Supplementary File 2 is the only string that was used across all databases, then the first full sentence on page 8 (“An example…”) should be updated to say so. A simple table could summarize these strings, databases, and results effectively. Doing so would also give reviewers (both like myself for this protocol and for the potential full manuscript) a good idea of if there is enough literature or ‘critical mass’ for a scoping review to be effective. | We agree that providing a comprehensive overview of the search strategy will improve transparency. In response, we have revised Supplemental File 2 to include the final search string and corresponding databases.We have also revised the manuscript text to clarify these changes (see Page 7).  As this manuscript presents a protocol, it is not necessary to include search results at this stage; however, these will be fully reported in the completed scoping review. |
| 2.4 The section ‘Charting the Data’ describes a standardized extraction form. The authors should if able include this form as a supplementary document. | We agree that including the data extraction form will improve transparency and clarity. In response, we have added the standardized extraction form as Supplemental File 3. We have also revised the manuscript text to reflect the inclusion of the new supplemental file (Page 9) |
| 2.5 The authors provide no plan for posting their data publicly upon completion of the review. The authors have also not registered with PROSPERO. | This manuscript presents a protocol, therefore no datasets have been generated and there are no data to share at this protocol stage. Regarding registration, scoping reviews are not required to be registered in PROSPERO, which is typically limited to systematic reviews. |
| 2.6 Throughout, I believe the in-text citations need to be in square rather than standard brackets for PLOS ONE. | The in-text citations have been updated to square brackets per PLOS ONE guidelines. |
| 2.7 Key terms (page 7) could be summarized in a table. For example, take each core concept as a column and include synonyms down the rows. Such a table would also help readers intuit how the search strings function (per the Boolean operators). | Key terms are now organized by core concept in Table 1 (Pages 7-8). |
| 2.8 Does Covidence have/need a citation? | The website for Covidence was added in parentheses. (Page 8) |
| 2.9 “Afterwards, the titles and abstracts of each included record will be independently screened by two of three reviewers. That is, three reviewers will participate in the title and abstract screening but each record will only be reviewed by two of the three reviewers.” – On page 8, this could be one sentence. This whole paragraph (‘Study Selection’, p. 8) reads a little confusing. | This paragraph has been revised. For example, the second sentence was removed to streamline the paragraph (Page 8-9) |
| 2.10 What happens if authors are unresponsive for those papers behind paywalls/that are inaccessible? (under ‘Study Selection’, p. 8) | Thank you for raising this question. The following sentence was added: “However, records will be excluded if the author(s) of the record are unresponsive to requests or if the record is otherwise inaccessible.” (Page 9) |
| 2.11 In ‘Inclusion and Exclusion Criteria’ on p. 8 & 9, many of the statements are redundant (e.g. “Also, records must be available in English” and “Records unavailable in English will also be excluded”). These criteria could be summarized as a list. It may also be worth mentioning that results can come from anywhere in the world (if that is the case). | Thank you for raising this concern. The ‘Inclusion and Exclusion’ criteria section was revised for redundancy and reformatted into a list. The possibility for results to come from anywhere in the world is also now included. (Page 9) |
| 2.12 Then, data from the key findings section of the extraction form will be coded inductively and independently by two reviewers. That is, no codebook will be developed. Instead, two reviewers will use open coding to code the extracted data. Afterwards, the three reviewers will meet to discuss the codes and resolve any discrepancies. – is there any particular reason why the authors are choosing to not use a codebook? If there are specific justifications for having each reviewer use open coding instead of something more standardized, I highly recommend including them explicitly. Otherwise, the choice appears somewhat arbitrary. | Thank you for raising this concern. We have now shared our rationale (with citation) for using an inductive approach without a codebook. Adding this rationale has helped to strengthen the quality and transparency of this section. Thank you, again, for your feedback! (Page 10) |
