# Supplementary material for: Animal-vehicle collisions, roadkilled animals, and human health: A scoping review protocol
Source: PLoS One. 2026 Aug 4;21(8):e0341107. doi: 10.1371/journal.pone.0341107 (PMC13436736; doi:10.1371/journal.pone.0341107)
Supplement: Peer review history

# Peer review: Decision Letter 0

**DOI:** 10.1371/journal.pone.0341107.r001

**Authors:** Vitor Paiva

11 Jun 2026

PONE-D-25-69050

Wildlife-vehicle collisions, roadkilled animals, and human health: A scoping review protocol

PLOS One

Dear Dr. Doherty,

Thank you for submitting your manuscript to PLOS ONE. After careful consideration, we feel that it has merit but does not fully meet PLOS ONE’s publication criteria as it currently stands. Therefore, we invite you to submit a revised version of the manuscript that addresses the points raised during the review process.

Please submit your revised manuscript by Jul 26 2026 11:59PM. If you will need more time than this to complete your revisions, please reply to this message or contact the journal office at plosone@plos.org. When you're ready to submit your revision, log on to https://www.editorialmanager.com/pone/ and select the 'Submissions Needing Revision' folder to locate your manuscript file.

Please include the following items when submitting your revised manuscript:

- A letter that responds to each point raised by the academic editor and reviewer(s). You should upload this letter as a separate file labeled 'Response to Reviewers'.
- A marked-up copy of your manuscript that highlights changes made to the original version. You should upload this as a separate file labeled 'Revised Manuscript with Track Changes'.
- An unmarked version of your revised paper without tracked changes. You should upload this as a separate file labeled 'Manuscript'.

If you would like to make changes to your financial disclosure, please include your updated statement in your cover letter. Guidelines for resubmitting your figure files are available below the reviewer comments at the end of this letter.

If applicable, we recommend that you deposit your laboratory protocols in protocols.io to enhance the reproducibility of your results. Protocols.io assigns your protocol its own identifier (DOI) so that it can be cited independently in the future. For instructions see: https://journals.plos.org/plosone/s/submission-guidelines#loc-laboratory-protocols. Additionally, PLOS ONE offers an option for publishing peer-reviewed Lab Protocol articles, which describe protocols hosted on protocols.io. Read more information on sharing protocols at https://plos.org/protocols?utm_medium=editorial-email&utm_source=authorletters&utm_campaign=protocols.

As the corresponding author, your ORCID iD is verified in the submission system and will appear in the published article. PLOS supports the use of ORCID, and we encourage all coauthors to register for an ORCID iD and use it as well. Please encourage your coauthors to verify their ORCID iD within the submission system before final acceptance, as unverified ORCID iDs will not appear in the published article. *Only*  the individual author can complete the verification step; PLOS staff *cannot*  verify ORCID iDs on behalf of authors.

We look forward to receiving your revised manuscript.

Kind regards,

Vitor Hugo Rodrigues Paiva, Ph.D.

Academic Editor

PLOS One

Journal Requirements:

When submitting your revision, we need you to address these additional requirements.

1. Please ensure that your manuscript meets PLOS ONE's style requirements, including those for file naming. The PLOS ONE style templates can be found at

https://journals.plos.org/plosone/s/file?id=wjVg/PLOSOne_formatting_sample_main_body.pdf and

https://journals.plos.org/plosone/s/file?id=ba62/PLOSOne_formatting_sample_title_authors_affiliations.pdf

2. When completing the data availability statement of the submission form, you indicated that you will make your data available on acceptance. We strongly recommend all authors decide on a data sharing plan before acceptance, as the process can be lengthy and hold up publication timelines. Please note that, though access restrictions are acceptable now, your entire data will need to be made freely accessible if your manuscript is accepted for publication. This policy applies to all data except where public deposition would breach compliance with the protocol approved by your research ethics board. If you are unable to adhere to our open data policy, please kindly revise your statement to explain your reasoning and we will seek the editor's input on an exemption. Please be assured that, once you have provided your new statement, the assessment of your exemption will not hold up the peer review process.

3. Please include captions for your Supporting Information files at the end of your manuscript, and update any in-text citations to match accordingly. Please see our Supporting Information guidelines for more information: http://journals.plos.org/plosone/s/supporting-information.

If the reviewer comments include a recommendation to cite specific previously published works, please review and evaluate these publications to determine whether they are relevant and should be cited. There is no requirement to cite these works unless the editor has indicated otherwise.

4. Please review your reference list to ensure that it is complete and correct. If you have cited papers that have been retracted, please include the rationale for doing so in the manuscript text, or remove these references and replace them with relevant current references. Any changes to the reference list should be mentioned in the rebuttal letter that accompanies your revised manuscript. If you need to cite a retracted article, indicate the article’s retracted status in the References list and also include a citation and full reference for the retraction notice.

[Note: HTML markup is below. Please do not edit.]

Reviewers' comments:

Reviewer's Responses to Questions

**Comments to the Author**

1. Does the manuscript provide a valid rationale for the proposed study, with clearly identified and justified research questions?

The research question outlined is expected to address a valid academic problem or topic and contribute to the base of knowledge in the field.

Reviewer #1: Yes

Reviewer #2: Yes

**********

2. Is the protocol technically sound and planned in a manner that will lead to a meaningful outcome and allow testing the stated hypotheses?

The manuscript should describe the methods in sufficient detail to prevent undisclosed flexibility in the experimental procedure or analysis pipeline, including sufficient outcome-neutral conditions (e.g. necessary controls, absence of floor or ceiling effects) to test the proposed hypotheses and a statistical power analysis where applicable. As there may be aspects of the methodology and analysis which can only be refined once the work is undertaken, authors should outline potential assumptions and explicitly describe what aspects of the proposed analyses, if any, are exploratory.

Reviewer #1: Partly

Reviewer #2: Yes

**********

3. Is the methodology feasible and described in sufficient detail to allow the work to be replicable?

Descriptions of methods and materials in the protocol should be reported in sufficient detail for another researcher to reproduce all experiments and analyses. The protocol should describe the appropriate controls, sample size calculations, and replication needed to ensure that the data are robust and reproducible.

Reviewer #1: Yes

Reviewer #2: Yes

**********

4. Have the authors described where all data underlying the findings will be made available when the study is complete?

The PLOS Data policy requires authors to make all data underlying the findings described in their manuscript fully available without restriction, with rare exception, at the time of publication. The data should be provided as part of the manuscript or its supporting information, or deposited to a public repository. For example, in addition to summary statistics, the data points behind means, medians and variance measures should be available. If there are restrictions on publicly sharing data—e.g. participant privacy or use of data from a third party—those must be specified.

Reviewer #1: No

Reviewer #2: No

**********

5. Is the manuscript presented in an intelligible fashion and written in standard English?

PLOS ONE does not copyedit accepted manuscripts, so the language in submitted articles must be clear, correct, and unambiguous. Any typographical or grammatical errors should be corrected at revision, so please note any specific errors here.

Reviewer #1: Yes

Reviewer #2: Yes

**********

6. Review Comments to the Author

Please use the space provided to explain your answers to the questions above and, if applicable, provide comments about issues authors must address before this protocol can be accepted for publication. You may also include additional comments for the author, including concerns about research or publication ethics.

You may also provide optional suggestions and comments to authors that they might find helpful in planning their study.

(Please upload your review as an attachment if it exceeds 20,000 characters)

Reviewer #1: The manuscript describes a scoping review protocol for understanding the various kinds of impacts of wildlife-vehicle collisions on humans. This is a relatively understudied aspect of roadkill, and I congratulate the authors for devising this study. I look forward to reading the results of the completed study, as it potentially has important repercussions for human health, and can also help argue for safer roads for wildlife.

The review protocol is well-written, and well-described. I have a few comments, specifically pertaining to the flow and readability of the text. Certain things need to be described and clarified, for the manuscript to be understood by the wider scientific readership.

Below are my detailed comments:

Abstract

Background: include physical (injury, fatality)

Search strategy: I suggest detailing the process of managing screening (in the methods in detail) instead of prescribing a paid software, which may not be accessible to some researchers trying to replicate the process.

Introduction:

Page 4, para 1,2: Are you including domestic animal collisions too? It would be prudent to specify this at the beginning (since you also use the term ‘roadkilled animals’). Although the term ‘WVC’ indicates the study is concentrated on wildlife species only, a lot of domestic/free-ranging animals are also killed on roads, and can affect humans similarly. Alternately, define WVC at the beginning (wild animal fatalities resulting from collisions with vehicles).

Para 2: I suggest reorganising the second paragraph to direct the narrative towards human-impacts of WVCs, from the relatively well-studied aspect i.e., ecological impacts.

Page 5, para 2: “prior reviews have examined WVCs and roadkilled animals more broadly” suggest defining ‘broad’ (mainly ecological, patterns etc).

Paras 2,3: I understand the rationale behind the review (to be conducted) and how the scoping process is designed to achieve that. I believe separating the two would make for easier reading and comprehension for readers, and strengthen the introduction. I suggest: first state the merits/novelty of the proposed review framework, and then the perceived benefits of the resulting review results (or vice versa).

Page 8 (Study Selection): same comment regarding the software. Consider excluding the name, except if it is central to your review process, or no other alternatives exist (including open-source or free ones).

Page 8 (inclusion and exclusion criteria): “Records may be cross-sectional or longitudinal.” needs clarification

Page 10: Please describe the rationale behind independent coding of findings by reviewers, and not based on agreed upon codes and pre-defined categories. If I understand correctly, ‘open coding’ means it is up to the reviewers to interpret and code findings independently. On what basis will the discrepancies be resolved? Are the categories and sub-categories pre-defined? This section needs a little clarification and restructuring. Can the authors define what ‘code’ and ‘categories’ mean (maybe through examples) for readers unfamiliar with content analysis?

Reviewer #2: Main review:

This manuscript describes a scoping protocol (to assess the literature landscape, rather than to systematically review it) to assess the impacts of wildlife-vehicle collisions and exposure to roadkill on various factors of human health. The authors will be focusing on physical, social, and emotional impacts in their review. The protocol follows PRISMA-SrC for reviews and the results will be descriptive of the distribution of the literature rather than a systematic review of what the literature describes (as is appropriate for a scoping review).

I believe that the proposed study would be important and of interest to sociologists, ecologists, and policymakers alike. The protocol is generally thorough and includes most of the necessary components (some are discussed by the authors but were not included in the submission). The citations all appear to be sound and properly formatted. I feel like the protocol does a decent job describing the study but can be tightened up in a few ways:

Major comments:

1. I am a little surprised at the sparseness of the citations regarding the ecological consequences of roadkill given the ubiquity and significance of roadkill around the world. I am not a roadkill expert by any means, but even a cursory search yields several other ecological reviews that seem relevant to the problem at hand. For example (amongst many others):

https://doi.org/10.1016/j.jtrangeo.2006.11.006

https://doi.org/10.1007/s10344-019-1357-4

https://doi.org/10.1111/brv.12942

https://doi.org/10.1371/journal.pone.0166941

I understand that the purpose of the proposed review is to focus on the human impacts (especially the psychological/emotional impacts) rather than ecological factors. However, I feel that a stronger engagement with the ecological literature would benefit the authors regarding the various avenues through which roadkill yields emotional impacts (and thus the various ways of categorizing their findings in a review). For example, roadkill may also be a vector for contaminants/disease which (1) could be a significant physical impact of interacting with roadkill and (2) creates an entirely different collection of stressors than those caused by an initial collision.

Especially given that the authors intend to use an open coding approach, the more familiarity and understanding of the possible categories various studies may fall under, the better. I would be far more confident in the eventual choices of the authors if there was a clearer demonstration of engagement with the literature at this stage.

2. If the scoping search has already been conducted (which is the case here), then I believe all of the search strings, their relevant databases, and the results at the time of the search should be reported, rather than the one example given in Supplemental File 2. If the string in Supplementary File 2 is the only string that was used across all databases, then the first full sentence on page 8 (“An example…”) should be updated to say so. A simple table could summarize these strings, databases, and results effectively. Doing so would also give reviewers (both like myself for this protocol and for the potential full manuscript) a good idea of if there is enough literature or ‘critical mass’ for a scoping review to be effective.

3. The section ‘Charting the Data’ describes a standardized extraction form. The authors should if able include this form as a supplementary document.

4. The authors provide no plan for posting their data publicly upon completion of the review. The authors have also not registered with PROSPERO.

Other minor comments:

1. Throughout, I believe the in-text citations need to be in square rather than standard brackets for PLOS ONE.

2. Key terms (page 7) could be summarized in a table. For example, take each core concept as a column and include synonyms down the rows. Such a table would also help readers intuit how the search strings function (per the Boolean operators).

3. Does Covidence have/need a citation?

4. “Afterwards, the titles and abstracts of each included record will be independently screened by two of three reviewers. That is, three reviewers will participate in the title and abstract screening but each record will only be reviewed by two of the three reviewers.” – On page 8, this could be one sentence. This whole paragraph (‘Study Selection’, p. 8) reads a little confusing.

5. What happens if authors are unresponsive for those papers behind paywalls/that are inaccessible? (under ‘Study Selection’, p. 8)

6. In ‘Inclusion and Exclusion Criteria’ on p. 8 & 9, many of the statements are redundant (e.g. “Also, records must be available in English” and “Records unavailable in English will also be excluded”). These criteria could be summarized as a list. It may also be worth mentioning that results can come from anywhere in the world (if that is the case).

7. Then, data from the key findings section of the extraction form will be coded inductively and independently by two reviewers. That is, no codebook will be developed. Instead, two reviewers will use open coding to code the extracted data. Afterwards, the three reviewers will meet to discuss the codes and resolve any discrepancies. – is there any particular reason why the authors are choosing to not use a codebook? If there are specific justifications for having each reviewer use open coding instead of something more standardized, I highly recommend including them explicitly. Otherwise, the choice appears somewhat arbitrary.

**********

7. PLOS authors have the option to publish the peer review history of their article (what does this mean?). If published, this will include your full peer review and any attached files.

If you choose “no”, your identity will remain anonymous but your review may still be made public.

**Do you want your identity to be public for this peer review?**  For information about this choice, including consent withdrawal, please see our Privacy Policy.

Reviewer #1: No

Reviewer #2: **Yes:** Eden J Forbes

**********

[NOTE: If reviewer comments were submitted as an attachment file, they will be attached to this email and accessible via the submission site. Please log into your account, locate the manuscript record, and check for the action link "View Attachments". If this link does not appear, there are no attachment files.]

To ensure your figures meet our technical requirements, please review our figure guidelines: https://journals.plos.org/plosone/s/figures

You may also use PLOS’s free figure tool, NAAS, to help you prepare publication quality figures: https://journals.plos.org/plosone/s/figures#loc-tools-for-figure-preparation.

NAAS will assess whether your figures meet our technical requirements by comparing each figure against our figure specifications.

# Author comment: Author response to Decision Letter 1

**DOI:** 10.1371/journal.pone.0341107.r002

30 Jun 2026

Thank you for the opportunity to revise our manuscript. We greatly appreciate the reviewers’ thoughtful and constructive feedback, which has helped us strengthen the clarity, precision, and overall contribution of the paper. We have carefully considered and addressed all comments and suggestions, making revisions throughout the manuscript where appropriate. Detailed responses to each reviewer’s feedback are outlined in the Response to Reviewers document, with corresponding changes noted in the text.

Attachment Submitted filename: Response to Reviewers.docx

# Editor report: Decision Letter 1

**DOI:** 10.1371/journal.pone.0341107.r003

**Authors:** Vitor Paiva

5 Jul 2026

Animal-vehicle collisions, roadkilled animals, and human health: A scoping review protocol

PONE-D-25-69050R1

Dear Dr. Doherty,

We’re pleased to inform you that your manuscript has been judged scientifically suitable for publication and will be formally accepted for publication once it meets all outstanding technical requirements.

Within one week, you’ll receive an e-mail detailing the required amendments. When these have been addressed, you’ll receive a formal acceptance letter and your manuscript will be scheduled for publication.

An invoice will be generated when your article is formally accepted. Please note, if your institution has a publishing partnership with PLOS and your article meets the relevant criteria, all or part of your publication costs will be covered. Please make sure your user information is up-to-date by logging into Editorial Manager at Editorial Manager® and clicking the ‘Update My Information' link at the top of the page. For questions related to billing, please contact billing support.

If your institution or institutions have a press office, please notify them about your upcoming paper to help maximize its impact. If they’ll be preparing press materials, please inform our press team as soon as possible -- no later than 48 hours after receiving the formal acceptance. Your manuscript will remain under strict press embargo until 2 pm Eastern Time on the date of publication. For more information, please contact onepress@plos.org.

Kind regards,

Vitor Hugo Rodrigues Paiva, Ph.D.

Academic Editor

PLOS One

Additional Editor Comments (optional):

Reviewers' comments:

# Editor report: Acceptance letter

**DOI:** 10.1371/journal.pone.0341107.r004

**Authors:** Vitor Paiva

PONE-D-25-69050R1

PLOS One

Dear Dr. Doherty,

I'm pleased to inform you that your manuscript has been deemed suitable for publication in PLOS One. Congratulations! Your manuscript is now being handed over to our production team.

At this stage, our production department will prepare your paper for publication. This includes ensuring the following:

* All references, tables, and figures are properly cited

* All relevant supporting information is included in the manuscript submission,

* There are no issues that prevent the paper from being properly typeset

You will receive further instructions from the production team, including instructions on how to review your proof when it is ready. Please keep in mind that we are working through a large volume of accepted articles, so please give us a few days to review your paper and let you know the next and final steps.

Lastly, if your institution or institutions have a press office, please let them know about your upcoming paper now to help maximize its impact. If they'll be preparing press materials, please inform our press team within the next 48 hours. Your manuscript will remain under strict press embargo until 2 pm Eastern Time on the date of publication. For more information, please contact onepress@plos.org.

You will receive an invoice from PLOS for your publication fee after your manuscript has reached the completed accept phase. If you receive an email requesting payment before acceptance or for any other service, this may be a phishing scheme. Learn how to identify phishing emails and protect your accounts at https://explore.plos.org/phishing.

If we can help with anything else, please email us at customercare@plos.org.

Thank you for submitting your work to PLOS ONE and supporting open access.

Kind regards,

PLOS ONE Editorial Office Staff

on behalf of

Dr. Vitor Hugo Rodrigues Paiva

Academic Editor

PLOS One
